# The Hard Problem of Consciousness and the Free Energy Principle

**DOI:** 10.3389/fpsyg.2018.02714

**Published:** 2019-01-30

**Authors:** Mark Solms

**Affiliations:** Department of Psychology, University of Cape Town, Cape Town, South Africa

**Keywords:** hard problem, consciousness, free energy, predictive processing, affect, Freud

## Abstract

This article applies the free energy principle to the hard problem of consciousness. After clarifying some philosophical issues concerning functionalism, it identifies the elemental form of consciousness as *affect* and locates its physiological mechanism (an extended form of homeostasis) in the upper brainstem. This mechanism is then formalized in terms of free energy minimization (in unpredicted contexts) where decreases and increases in expected uncertainty are felt as pleasure and unpleasure, respectively. Emphasis is placed on the reasons why such existential imperatives *feel like something* to and for an organism.

I recently published a dense article on this topic (Solms and Friston, 2018)—a sort of preliminary communication—which I would like to expand upon here, in advance of a book-length treatment to be published under the title *Consciousness Itself* (Solms, in press). Since this is a psychoanalytic journal, I will supplement my argument with cross-references to Freud's views on these themes. Readers with a mathematical background will benefit from a close reading of Solms and Friston (2018) in conjunction with this paper, which is aimed primarily at a psychologically educated readership.

My argument unfolds over four sections, of unequal length. The first addresses some philosophical issues pertaining to dual-aspect monism in relation to the hard problem. The second reconsiders the anatomical localization of consciousness (the so-called neural correlate of consciousness or NCC) in the cerebral cortex. In consequence, it reconceptualizes the functional roles of the “level” vs. “contents” of consciousness. The third and most important section explains the dual aspects of consciousness (its physiological and psychological manifestations) in formal mechanistic terms, in relation to the imperatives of free energy minimization. The fourth section briefly pursues some implications of this formulation for the cognitive neuroscience of consciousness, in relation to memory consolidation and reconsolidation.

## The Problem With The Hard Problem

### Does the Brain Produce the Mind?

The original statement of the hard problem, as formulated by David Chalmers, is put like this:

It is undeniable that some organisms are subjects of experience. But the question of how it is that these systems are subjects of experience is perplexing. Why is it that when our cognitive systems engage in visual and auditory information-processing, we have visual or auditory experience: the quality of deep blue, the sensation of middle C? How can we explain why there is something it is like to entertain a mental image, or to experience an emotion? It is widely agreed that experience arises from a physical basis, but we have no good explanation of why and how it so arises. Why should physical processing give rise to a rich inner life at all? It seems objectively unreasonable that it should, and yet it does (Chalmers, 1995).

A shorter statement of the problem goes like this: “How and why do neurophysiological activities *produce* the “experience of consciousness?” (Chalmers, 1996, emphasis added). John Searle says something similar: “How exactly do neurobiological processes in the brain *cause* consciousness?” (Searle, 2017, p. xiii, emphasis).

The starting point of the argument I shall set out here is that the brain does not “produce” or “cause” consciousness. Formulating the relationship between the brain and the mind in causal terms makes the hard problem harder than it needs to be. The brain does not produce consciousness in the sense that the liver produces bile, and physiological processes do not cause—or become or turn into—mental experiences through some curious metaphysical transformation.

When I wake up in the morning and experience myself (my mind) to exist, and then confirm in the mirror that I (my body) do indeed exist, I am simply realizing the same thing from two different *observational perspectives* (first-person and second-person perspectives). Asking how my body produces my mental experience is like asking how lightning causes thunder.

This is the dual-apect monist position on the mind/body problem^1^. There can of course be no question of determining a “correct” metaphysical starting point, but the dual-aspect monist position—which is the starting point of my argument—raises an interesting philosophical question. If body and mind are two appearances (aspects) of the same underlying thing, then what stuff is the underlying thing made of? In other words, using the analogy of thunder and lightning, what is the metapsychological^2^ equivalent of “electricity” (i.e., the thing that gives rise to thunder and lightning, both)?

This question requires one to clarify what we mean (ontologically) by terms like “physical basis,” “physical processing,” “neurophysiological activities,” and “neurobiological processes”—terms which turn out to be surprisingly ambiguous. If physiological phenomena—like their mental correlates—are appearances, then their basis must be something non-physiological.

Let us approach the question by way of an example. If the internal experience of having a memory and the neuronal assemblage embodying that same memory (pictured externally, through optogenetics, for example) are two realizations of a single underlying thing, then what is “memory” itself made of? The answer is that it is abstracted from both manifestations. Memory is not a stuff; it is a *function*. We describe functions in terms of their underlying lawful mechanics, not their appearances^3^. The laws are inferred from the regularities we observe; they *explain* the appearances.

There are of course both psychological and physiological accounts of the functions of memory; but the mechanism a dual-aspect monist is looking for must be sufficiently deep to account equally for both of its observable manifestations—psychological and physiological. In the above example: if we explain the experience of remembering in psychological terms and the activation of the neuronal assemblage (and associated cellular processes) in physiological terms, then our functional inferences are too superficial, and an “explanatory gap” will appear between them (Levine, 1983). Accordingly, one must infer laws which are abstracted equally from the two phenomenal surfaces, sufficiently deeply to underpin the psychological *and* physiological accounts^4^.

This is not difficult to do. Consider, for example, short-term memory (STM). Miller's law states that human beings are capable of holding seven-plus-or-minus-two units of information in working memory at any one point in time. This is an abstraction derived both from the (psychological) experience of trying to hold more than seven things in mind and from observing the correlated (physiological) synaptic dynamics of STM traces (Mongillo et al., 2008). The same applies to Ribot's law, concerning the temporal gradient of long-term memory (LTM), which underpins both the psychological and physiological phenomena of memory consolidation over time (Kandel et al., 2012). These laws concern the behavior of an abstracted function, which is (in itself) both psychological and physiological. Ultimately, in all sciences, we aspire to reduce such laws to formalized algorithms—to mathematics—the ideal of third-person abstraction^5^.

That is why terms like “physical basis” and “neurobiological processes,” etc., are surprisingly ambiguous in relation to mental functions. They suggest asymmetrical (i.e., overly superficial) functional concepts which can explain only the neurological side of the neuro/psychological equation—thereby leaving an explanatory gap.

But before one can identify the functional laws underpinning the regularities of both conscious experience *and* its neural correlates, one faces a further hurdle.

### Is Consciousness Just Another Cognitive Function?

Chalmers insists that consciousness cannot be explained in functional terms. He claims that reducing consciousness (as we experience it) to a functional mechanism will *never* solve the hard problem:

The easy problems are easy precisely because they concern the explanation of cognitive abilities and functions. To explain a cognitive function, we need only specify a mechanism that can perform the function. The methods of cognitive science are well-suited for this sort of explanation, and so are well-suited to the easy problems of consciousness. By contrast, the hard problem is hard precisely because it is not a problem about the performance of functions. The problem persists even when the performance of all the relevant functions is explained … What makes the hard problem hard and almost unique is that it goes *beyond* problems about the performance of functions. To see this, note that even when we have explained the performance of all the cognitive and behavioral functions in the vicinity of experience … there may still remain a further unanswered question: *Why is the performance of these functions accompanied by experience?* A simple explanation of the functions leaves this question open … Why doesn't all this information-processing go on “in the dark,” free of any inner feel? (Chalmers, 1995).

In the passage just quoted, Chalmers draws attention to the fact that consciousness is not just a cognitive function. It is easy to agree with him. All cognitive functions (such as memory) are not intrinsically conscious. There does not have to be “something it is like” to remember. It is well-established that learning and memory can exert their effects without any “inner feel”; and the same applies to perception. Hence the title of (Kihlstrom's, 1996) celebrated review article: “Perception without Awareness of What Is Perceived, Learning Without Awareness of What Is Learned.” The only exception to the rule is precisely what needs to be explained: namely the *conscious part* of cognition—the part that is left over when the performance of all the relevant functions is explained.

Why is experience left unexplained, even when we have explained the performance of all the relevant cognitive functions in its vicinity? Some philosophers assert it is because “consciousness has a first person or subjective ontology and so cannot be reduced to anything that has third-person or objective ontology” (Searle, 1997, p. 212). The hard problem would be trivial if all it boils down to is the fact that your own personal experience, here and now, is not reducible to human experience in general. All one would need to do, then, to solve the problem, would be to take the experiences of lots of individuals, average them, find the common denominator, and explain *that* in functional terms. Psychologists do this sort of thing all the time. But Chalmers is not asking something so trivial. He writes:

Why is it that when electromagnetic waveforms impinge on a retina and are discriminated and categorized by a visual system, this discrimination and categorization is experienced as a sensation of vivid red? We know that conscious experience *does* arise when these functions are performed, but the very fact that it arises is the central mystery. There is an *explanatory gap* (a term due to Levine, 1983) between the functions and experience, and we need an explanatory bridge to cross it. A mere account of the functions stays on one side of the gap, so the materials for the bridge must be found elsewhere (Chalmers, 1995).

Leaving aside his apparent conflation of two different kinds of explanatory gap (between experience and physiology on the one hand and experience and function on the other) it now becomes apparent why Chalmers believes that even the latter gap is unbridgeable. *He is focusing on the wrong function*. An explanation of experience will never be found in the function of vision—or memory, for that matter—or in any function that is not inherently experiential.

The function of experience cannot be inferred from perception and memory, but it *can* be inferred from feeling. There is not necessarily “something it is like” to perceive and to learn, but who ever heard of an unconscious feeling—a feeling that you cannot feel?^6^ If we want to identify a mechanism that explains the phenomena of consciousness (in both its psychological and physiological aspects) we must focus on the function of feeling—the technical term for which is “affect.” That is why it is easy to agree that consciousness is not just another cognitive function. Cognition has long been distinguished from affect, and for good reason^7^.

This focus on affect is far from arbitrary.

## In The Beginning Was The Affect

### Is Consciousness a Cortical Function?

The massive effort in recent times to identify the NCC—*The Scientific Search for the Soul*, as Francis Crick (1994) memorably called it—used vision as its model example. This was justified by the fact that the details of visual processing are better understood than those for any other modality of consciousness.

Crick's strategy was that the NCC for vision should be generalizable to other forms of consciousness. His reasoning was simple: it must be possible to isolate something going on somewhere in the visual brain when you are seeing consciously which is absent when you are seeing unconsciously, and this is the NCC for vision. Closer study of this NCC (whatever it turns out to be: activation of a specific type of neuron, or a specific neural network, or a specific frequency band, etc.) should eventually reveal how and why visual consciousness arises.

In Chalmers's opinion, Crick's strategy is only capable of solving the easy (correlational) part of the mind/body problem; it cannot solve the hard (causal) part. There are at least three further problems with Crick's strategy.

The first is that there cannot be any objects of consciousness without a *subject* of consciousness. You cannot experience objects (visually or otherwise) unless *you* are there to experience them. This calls into question whether the essence of conscious experience resides in any perceptual modality. What if the NCC resides in the thing which binds the objects of conscious perception—in the perceiver rather than the perceptions?

This problem need not be fatal for Crick, if it turns out that experiencing arises from some aggregate of, or some interaction between, etc., the various types of perception—as some theorists claim it does. The experiencing subject need not take the form of a homunculus; it might be distributed over the cortex and emerge through a mechanism akin to trans-cortical “association.” That is how the nineteenth century German anatomists saw it, when they first formulated the cortico-centric conception of consciousness on the model of seventeenth and eighteenth century British empiricist philosophies of mind (see Meynert, 1884).

This leads to a second problem with Crick's strategy. When Munk (1878, 1881) identified occipital cortex as the locus of the mental aspect of vision (which, importantly, he—like Meynert and the British empiricists—equated with the capacity to form visual “memory images” or “ideas,” as opposed to mere sensations) it seemed reasonable enough to generalize the principle—the principle that the cortex is the organ of the “mind” *so defined*—to the other modalities of perception^8^. The ensuing experimental findings confirmed the validity of this generalization (e.g., ablation of auditory cortex [in dogs, Munk's model species] produced “mind deafness,” just as occipital lesions caused “mind blindness”—which was subsequently also confirmed in human clinical cases; see Solms et al., 1996).

If we equate mind with memory images (and the associations between them) then it comes as no surprise to learn that, when Munk's contemporaries ablated the whole cortex, the animals did not fall into *coma*; instead, they became *amnestic* (see Meynert, 1884, Chapter 3, for review). Subsequent studies have confirmed this observation in numerous animal species (e.g., Huston and Borbely, 1974). Consciousness persists in the absence of cerebral cortex, as does volitional behavior. As Damasio and Carvalho (2013, p. 147) put it: “Decorticated mammals exhibit a remarkable persistence of coherent, goal-oriented behavior that is consistent with feelings and consciousness”.

The same facts are observed in congenitally decorticate (hydranencephalic) human beings. In view of the importance of this for our topic, I will cite a lengthy description:

In the setting of the home environment upon which these medically fragile children are crucially dependent, they give proof of being not only awake, but of the kind of responsiveness to their surroundings that qualifies as conscious by the criteria of ordinary neurological examination (Shewmon et al., 1999). The report by Shewmon and colleagues is the only published account based upon an assessment of the capacities of children with hydranencephaly under near optimal conditions, and the authors found that each of the four children they assessed was conscious. […] To supplement the limited information available in the medical literature on the behavior of children with hydranencephaly, I joined a worldwide internet self-help group formed by parents and primary caregivers of such children. Since February of 2003 I have read more than 26,000 e-mail messages passing between group members. Of these I have saved some 1,200 messages containing informative observations or revealing incidents involving the children. In October 2004 I joined five of these families for 1 week as part of a social get-together featuring extended visits to DisneyWorld with the children, who ranged in age from 10 months to 5 years. I followed and observed their behavior in the course of the many private and public events of that week, and documented it with 4 h of video recordings. My impression from this first-hand exposure to children with hydranencephaly confirms the account given by Shewmon and colleagues. These children are not only awake and often alert, but show responsiveness to their surroundings in the form of emotional or orienting reactions to environmental events […] They express pleasure by smiling and laughter, and aversion by “fussing,” arching of the back and crying (in many gradations), their faces being animated by these emotional states. A familiar adult can employ this responsiveness to build up play sequences predictably progressing from smiling, through giggling, to laughter and great excitement on the part of the child. The children respond differentially to the voice and initiatives of familiars, and show preferences for certain situations and stimuli over others, such as a specific familiar toy, tune, or video program, and apparently can even come to expect their regular presence in the course of recurrent daily routines. Though behavior varies from child to child and over time in all these respects, some of these children may even take behavioral initiatives within the severe limitations of their motor disabilities, in the form of instrumental behaviors such as making noise by kicking trinkets hanging in a special frame constructed for the purpose (“little room”), or activating favorite toys by switches, presumably based upon associative learning of the connection between actions and their effects. Such behaviors are accompanied by situationally appropriate signs of pleasure or excitement on the part of the child, indicating that they involve coherent interaction between environmental stimuli, motivational-emotional mechanisms, and bodily actions […] The children are, moreover, subject to the seizures of absence epilepsy. Parents recognize these lapses of accessibility in their children, commenting on them in terms such as “she is off talking with the angels,” and parents have no trouble recognizing when their child “is back.” […] The fact that these children exhibit such episodes would seem to be a weighty piece of evidence regarding their conscious status (Merker, 2007, p. 79).

“Associative learning of the connection between actions and their effects” does not imply the experience of “memory images,” but one must surely conclude that experience itself is not a cortical function. The ABCs of behavioral neuroscience demand that if a function (or critical component function) is localized in a particular structure, then ablation of that structure must result in loss of that function. In the case of consciousness in relation to the cerebral cortex, this critical test is failed.

I am aware that some readers will wonder about the above usage of the term “consciousness” (i.e., in what sense are these animals and children “conscious”); and they might invoke the epistemological problem of other minds (how do we *know* they are conscious). Before addressing these questions, let us consider a third problem with the cortico-centric approach.

The third problem is that there is a brain structure which *does* pass the critical test just mentioned. This structure is located not in the cortex but the brainstem.

The seminal observations were made in cats by Moruzzi and Magoun (1949), and confirmed in humans by Penfield and Jasper (1954). Consciousness is obliterated by focal lesions of the brainstem core^9^—in a region conventionally described as the extended reticulothalamic activating system (ERTAS). Recent findings indicate that the smallest lesions within the brainstem which cause total loss of consciousness (i.e., coma) are located in or near the parabrachial nuclei of the pons (Parvizi and Damasio, 2003; Golaszewski, 2016).

Why, then, did Crick and his followers not look for the NCC in the brainstem? The answer is: for reasons of convention. After Moruzzi & Magoun failed to confirm a major prediction arising from the classical theory, namely that deprivation of sensory inputs to cortex should result in loss of consciousness (e.g., sleep)^10^, they did not abandon the theory; instead they introduced a distinction between the “contents” and “level” of consciousness. This saved the old theory. The contents (the *qualia* of consciousness) were thereby still assigned to the cortex, and a new level-regulating function (the *quantity* of arousal or wakefulness, measured on a 15-point scale) was assigned to the ERTAS.

This assignment continues to this day. Crick's closest collaborator, Christof Koch, therefore says of the deep brainstem nuclei that “they are *enablers* [of consciousness] but not content-providers” (Koch, 2004, p. 93, emphasis added). This takes us back to the question asked above: in what sense are decorticate animals and children conscious? Do they display *blank* wakefulness, devoid of content and quality, or is there “something it is like” to be them?

### Does it Feel Like Something to be Awake?

The conclusion of the argument being set out here may be stated in advance: the so-called level of consciousness is a function of variational *free energy*. Free energy in thermodynamic terms entails *entropy*, which in information-theoretic terms is *surprisal* (and *uncertainty*), which in neurophysiological terms is *arousal* (see Solms and Friston, 2018) Arousal underpins *wakefulness*. Later, these equivalencies will enable us to approach the Helmholtzian ideal of describing “the specific way or form of the action [of consciousness] by means of the physical-mathematical method.”^11^ As Pfaff (2006) says: “Because CNS arousal depends on surprise and unpredictability, its appropriate quantification depends on the mathematics of information” (p. 13).

The question at hand concerns the nature of the “consciousness” displayed by decorticate animals and children. Consistent with what Damasio and Carvalho (2013) said about animals, and with Shewmon, Holmse and Byrne's (1999) findings, Merker (2007) observed that hydranencephalic children show “emotional or orienting reactions to environmental events.” Moreover, “they express pleasure by smiling and laughter, and aversion by ‘fussing,’ arching of the back and crying (in many gradations), their faces being animated by these emotional states.” The states include “smiling, through giggling, to laughter and great excitement on the part of the child.” These children also “show preferences for certain situations and stimuli over others.” And their “behaviors are accompanied by situationally appropriate signs of pleasure or excitement.”

One surely must conclude that it does feel like something to be these children. By any reasonable standard^12^, one would have to accept that they—like decorticate animals—show *basic emotions*. In fact, decorticate animals display excessive emotionality (Huston and Borbely, 1974), as do human patients who suffer damage to the cortical structures that exert inhibitory control over the ERTAS and limbic system (Harlow, 1868).

These observations may be linked with the fact that deep brain stimulation (DBS) of centrencephalic structures, such as the ERTAS and periaqueductal gray (PAG), and of the limbic circuits arising from them, generates powerful affective responses (see Panksepp, 1998, for detailed review). Importantly, in relation to the question concerning how we know these patients are conscious: in DBS of human beings, they declare these subjective states in words (e.g., Blomstedt et al., 2007). Within the confines of the epistemological problem of other minds (whereby one can never know for certain whether anyone other than oneself is conscious) there can be no higher standard of proof for the inference that upper brainstem and limbic circuits generate affects^13^

This conclusion is further supported by the fact that drugs acting on the neuromodulators sourced in the ERTAS nuclei (serotonin, dopamine, noradrenaline, acetylcholine) have powerful effects on mood and anxiety, etc.—which is why they represent the mainstay of psychopharmacology today (Meyer and Quenzer, 2005). In other words, most psychotropic medications act via the ERTAS.

It is legitimate to say that affects are *generated* in these subcortical structures for the reason that the same effects can be observed in the absence of cortex. This contradicts the prevailing view that these nuclei merely “enable” the cortex to feel (Koch, 2004). It is noteworthy in this regard that patients with total destruction of the very structures which are specifically identified by cortico-centric theorists of affect—namely the prefrontal lobes and insula (e.g., Craig, 2009; LeDoux and Brown, 2017)—not only report preserved feeling states, but, as mentioned already, they display excessive emotionality (see Damasio et al., 2012)^14^.

Although many cognitive scientists still must be weaned of the view that the cerebral cortex is the seat of consciousness (see Panksepp et al., 2016, for a lengthy discussion of this controversy), the weight of evidence for the alternative view that the arousal processes generated in the upper brainstem and limbic system feel like something in and of themselves, is now overwhelming. Coupled with the huge body of evidence suggesting that cortical (cognitive) functions are *not* intrinsically conscious (see Bargh and Chartrand, 1999, for review) one is led to the conclusion that the classical German anatomists were right: the cortex is merely a repository of “memory images.” Cortex evidently provides “random-access memory” space (Solms and Panksepp, 2012, Ellis and Solms, 2018). This conclusion is consistent with the radical plasticity of cortex; so much so that the right hemisphere can take over the functions of the left, entirely, if it is removed early enough (Pulsifer et al., 2004); and when the optic nerve is redirected to auditory cortex, it learns to see (Sharma et al., 2000).

This line of thinking will be extended in section ‘Consciousness Arises Instead of a Memory-Trace,’ where it is argued that cortex *stabilizes* consciousness rather than generates it; i.e., that cortical functioning binds affective arousal, and thereby transforms it into conscious cognition.

It is undeniable that a hierarchical dependency relation exists between the cortical type of consciousness and the upper brainstem type. This is not a controversial claim; it is precisely what is meant by the conventional assertion that the ERTAS “enables” consciousness. In the absence of brainstem arousal there cannot be cortical consciousness, but the converse does not apply. Since these simple facts meet the gold standard for parsing neuropsychological functions—namely the principle of “double dissociation” (Teuber, 1955; see footnote 13)—we must conclude that consciousness is generated in the upper brainstem.

If core brainstem consciousness is the primary type, then consciousness is fundamentally *affective* (see Panksepp, 1998; Solms, 2013; Damasio, 2018). The arousal processes that produce what is conventionally called “wakefulness” constitute the experiencing subject. In other words, *the experiencing subject is constituted by affect*.

This reformulation of elemental consciousness has major ramifications for its functional mechanism, underscoring the conclusions reached at the end of section ‘The Problem With The Hard Problem’. It is perfectly reasonable to ask why visual information-processing doesn't go on in the dark, without any inner feel, but it is perverse to ask why affective arousal doesn't do so. How can affective arousal (i.e., the arousal of feeling) go on without any inner feel?

### Why Do We Feel?

Current theoretical efforts to answer this question were initiated by Damasio (1994), who identified feeling with registering states of the body—within a biological scale of values—whereby pleasurable vs. unpleasurable feelings register improving vs. deteriorating chances of survival and reproductive success^15^. On Damasio's theory, that is why we feel. His theory was substantially enhanced when he incorporated (Panksepp, 1998) findings to the effect that feelings are generated not in the cortex but the brainstem (and limbic system; see Damasio, 2010) and that the circuits in question do not register only here-and-now states (or “as if” states; Damasio, 1994) of the autonomic and sensory body, but also intrinsic brain states: brain systems for instincts^16^ like attachment, rage and play (see Damasio, 2018). The shift downward to the brainstem enabled Damasio (like Panksepp before him) to recognize that *the elemental form of consciousness is an extremely primitive function*. My own contribution to these theoretical efforts came relatively late in the day (Solms and Panksepp, 2012) and they revolved mainly around the precise relationship between homeostasis^17^ and feeling (Solms, 2013, 2018a; Solms and Friston, 2018).

To be clear: I do not claim (and nor did Panksepp or Damasio)^18^ that feeling arises from homeostasis in and of itself. I do not believe that thermostats are conscious. I do not even claim that all living creatures are conscious (although all living creatures are homeostatic). Even in human beings, homeostatic mechanisms which are totally devoid of consciousness are operative. The regulation of blood pressure is a clinically notorious example. In fact, one may go much further: like Freud (and just about everyone else these days) I do not claim that all human *mental* functions are conscious. This has important implications for philosophers like Nagel and Chalmers, who sometimes forget that “subjectivity” and “consciousness” are not synonymous words (This fact is especially problematical for Chalmers's panpsychism; see Chalmers, 1995, 1996).

What I am claiming is something else: feeling enables complex organisms to register—and thereby to regulate and prioritize through thinking and voluntary action—deviations from homeostatic settling points *in unpredicted contexts*. This adaptation, in turn, underwrites learning from experience. In predictable situations, organisms may rely on automatized reflexive responses (in which case, the biologically viable predictions are made through natural selection and embodied in the phenotype; see Clark, 2016). But if the organism is going to make plausible *choices* in novel contexts (cf. “free will”) it must do so via some type of here-and-now assessment of the relative *value* attaching to the alternatives (see Solms, 2014).

Crucially, in this process, the organism must stay “ahead of the wave” of the biological consequences of its choices (to use the analogy that gave Andy Clark's (2016) book its wonderful title: *Surfing Uncertainty*):

To deal rapidly and fluently with an uncertain and noisy world, brains like ours have become masters of prediction—surfing the waves of noisy and ambiguous sensory stimulation by, in effect, trying to stay just ahead of the place where the wave is breaking (p. xiv).

The proposal on offer here is that this imperative *predictive* function—which bestows the adaptive advantage of enabling organisms to survive in novel environments—is performed by feeling (see section ‘To Be Precise’ below for clarification of the pivotal role of *context* in the prioritization of affects, and thereby the “flavoring” of consciousness). On the present proposal, this is the causal contribution of qualia (see Solms and Friston, 2018).

Affective qualia are accordingly claimed to work like this: deviation away from a homeostatic settling point (increasing uncertainty) is felt as unpleasure, and returning toward it (decreasing uncertainty) is felt as pleasure^19^. There are many types (or “flavors”) of pleasure and unpleasure in the brain (Panksepp, 1998)^20^. The type identifies the need at issue, which enables the organism to minimize computational complexity (i.e., to focus on the matter at hand—rather than its organismic state as a whole—and thereby to minimize metabolic expenditure; see Solms and Friston, 2018, footnote 7). All needs cannot be felt at once. The prioritization of needs—i.e., the determination as to which need will be felt—must obviously depend crucially upon *context* (i.e., needs in relation to other needs, and needs in relation to opportunities)^21^. Feeling is therefore extended onto exteroception (i.e., it is contextualized: “I feel like this about *that*”) and transformed into cognitive consciousness (i.e., it is “bound”; see section ‘Consciousness Arises Instead of a Memory-Trace’). This in turn gives rise to voluntary action—and what we loosely call *thinking*—and, over longer time-scales, to learning from experience (Thinking, as Freud taught us, entails virtual action rather than real action, and thereby saves lives)^22^.

Consciousness (thus defined) is a biological imperative; it is the vehicle whereby complex organisms monitor and maintain their functional and structural integrity in unknown situations. The inherently subjective and qualitative nature of this auto-assessment process explains “how and why” it [consciousness] feels like something to the organism, for the organism (cf. Nagel, 1974). Specifically, increasing uncertainty in relation to any biological imperative *just is* “bad” from the (first-person) perspective of such an organism—indeed it is an existential crisis—while decreasing uncertainty *just is* “good.” This provides a very important clue as to how the “hard problem” may be solved. Consciousness adaptively determines which uncertainties must be felt (i.e., prioritized) in any given context. In short, consciousness is *felt uncertainty*. We will see shortly how and why the first person perspective arises.

At this point, however, we must confront what philosophers term the “conceivability problem.”

The function I have just described could conceivably be performed by non-conscious “feelings” (cf. philosophical zombies)—if evolution had found another way for living creatures to pre-emptively register and prioritize (to themselves and for themselves) such inherently qualitative existential dynamics in uncertain contexts. But the fact that something can conceivably be done differently doesn't mean that it is not done in the way that it is in the vertebrate nervous system. In this respect, consciousness is no different from any other biological function. Ambulation, for example, does not *necessarily* require legs (As Jean-Martin Charcot said: “Theory is good, but it doesn't prevent things from existing'; Freud, 1893, p. 13). It seems the conceivability argument only arose in the first place because we were looking for the NCC in the wrong place. One suspects the problem would never have arisen if we had started by asking how and why feelings (like hunger) arise in relation to the exigencies of life, instead of why experience attaches to cognition.

In the next section, I will reduce the function of consciousness to its formal essence. But I want to conclude the present section with a brief description of its anatomical realization:

Body-monitoring nuclei in the spinal cord (dorsal root ganglia), upper brainstem and diencephalon (e.g., solitary nucleus, area postrema, parabrachial nucleus, circumventricular organs, and hypothalamus) can only go so far in terms of meeting endogenous needs through internal (autonomic) adjustments. Beyond that limit, *external* action is called for. At that point, autonomic reflexes become *drives*. That is, interoceptive (mainly medial hypothalamic) “need detectors” trigger not only autonomic reflexes but also—following the crucial prioritization process performed by the midbrain “decision triangle” (see footnote 21 above)—feelings of hunger, thirst, etc. Through a final common pathway of ERTAS arousal these drives typically^23^ trigger dopaminergically-mediated “foraging” behaviors (viz., the behaviors that Panksepp (1998) calls “SEEKING” and Berridge (1996) calls “wanting”). Foraging reflects a phylogenetically determined prediction, namely the prediction that whatever I need will be found out there in the world. The difference between Panksepp's “SEEKING” (i.e., objectless drive) and Berridge's “wanting” (i.e., goal-oriented motivation) reflects the influence of learning upon the primary instinctual mechanism of desire—whereby affective SEEKING becomes cognitive “wanting” (through need/satisfaction matching)^24^. This facilitates the formation of LTM cause/effect relations between particular needs and their adequate aims and objects, which in turn yields the iterative “reward prediction error” cycle that codes ongoing learning from experience (see Schultz, 2016).

Fortunately, living organisms are not required to learn everything about the world from scratch. Each phenotype is endowed with innate predictions concerning biologically significant situations it is certain to encounter^25^. Panksepp (1998) terms these “emotional” and “sensory” affects (but it is important to recognize that the word “affect” is only justified to the extent that the relevant instinctual and reflexive predictions are felt, i.e., to the extent that they yield residual uncertainties, which require choice and learning from experience). Examples of “emotional” affects (each of which is marked by its own command neuromodulators and receptor types) are fear, rage, attachment and play; and examples of such “sensory” affects are pain, surprise and disgust (see Panksepp, 1998). Fear behaviors (freezing and fleeing), for example, are innate predictions; but each individual has to learn *what* to fear and *what else* might be done in response. What vertebrates do to meet their needs always consists in a combination of innate and learned behaviors.

The residual uncertainty (unmet needs—i.e., unsolved problems—of various types) arising from each such cycle of behavior is auto-evaluated, in the manner described above, by mechanisms located mainly in the PAG—the terminus of all affective circuitry^26^. Merker (2007) accordingly describes the PAG as part of a “synencephalic bottleneck,” where perception, action and affect come together, and choices are made as to “what to do next^27^.” (It is important to recognize that the terminal location of the PAG in the cycle just described renders it *functionally* “supra-cortical,” notwithstanding the fact that it is *anatomically* sub-cortical; see Merker, 2007). PAG activity, then, results in revised perception/action selection, via ERTAS (and more specific higher limbic) neuromodulatory adjustments. This is how simple feeling becomes “feeling *about that*^28^.”

Note that the evaluation cycle just described entails ongoing assessment of environmental events *and* the internal milieu (via body monitoring nuclei)—both of which are “external” to the nervous system—although, for obvious biological reasons, internal uncertainties will almost always trump external ones (Imagine the consequences of back-ranking changes in oxygenation or hydration or thermoregulation). That is why consciousness is quintessentially affective.

## To Be Precise

### How Does Homeostasis Arise?

If consciousness arises through a homeostatic mechanism, as the above physiological^29^ considerations suggest, then a lot rides on the question: how does homeostasis arise? The answer to this question should lead to the abstraction we are looking for (i.e., the abstraction that transcends psychological and physiological “appearances”).

According to Friston (2013) the answer is *free-energy minimization*. For self-organizing systems—including all living things, like us—to exist, they must *resist entropy* (quantified as free energy, but see below for the important role of precision weighting)^30^. That is, self-organizing systems can only persist over time by occupying “preferred” states—as opposed to being dispersed over all possible states, and thereby dissipating. This is a fundamental precondition of life—and indeed any self-organization. We need not concern ourselves here with how life arises. However, grounding the mechanism of consciousness in the essential prerequisites for life is not a bad starting point, since it is generally assumed that all conscious things are alive—although not all living things are conscious.

For a system to resist entropy, three conditions must be met: (i) There must be a boundary which separates the internal and external states of the system, and thereby insulates the system from the world. Let's call the former states “the system” and the latter states “the not-system”—rather than “the world,” for reasons that will soon be explained. (ii) There must be a mechanism which registers the influence of dissipative external forces—i.e. the free energy. Let's call this mechanism the “sensory states” of the system. (iii) There must be a mechanism which counteracts these dissipative forces—i.e. which binds the free energy. Let's call this mechanism the “active states” of the system, such as motor and autonomic reflexes^31^.

According to Friston (2013), these functional conditions—which enable self-organizing systems to exist and persist over time—emerge naturally (indeed necessarily) within any ergodic^32^ random dynamical system that possesses a *Markov blanket*^33^. This blanket establishes the boundary conditions above and is a probabilistic construct that depends upon what influences what (and what doesn't influence what). The Markov rules of causal influence provide the prerequisite (i) separation between the system and the not-system (i.e., the blanket itself), and equip the former with (ii) receptor capacities (the sensory states of the blanket) and (iii) effector capacities (the active states of the blanket). It is important to recognize that these sensory and active capacities are properties of the blanket—not of the states they interact with—which implies that the system insulated by a Markov blanket can only “know” states of the not-system *vicariously*. In other words, external states can only be “inferred” by the system—on the basis of “sensory impressions” upon the Markov blanket.

In fact, it is *essential* for external states to be inferred by the system if dissipative forces are to be resisted. This implies that the system must incorporate a *model* of the world, which then becomes the basis upon which it acts. Such models—like all models—are imperfect things. They can (and must) be improved in the light of unfolding evidence. In other words, the inferences the model generates for the system about conditions outside (inferences formed on the basis of the sensory consequences of its actions) take the form of predictions, and these predictions must be constantly tested and revised^34^. Thus, perception and action entail ongoing processes of *hypothesis testing*, whereby the system updates its model—its “beliefs^35^”—over time. This imperative of negentropic self-organizing systems is, in a nutshell, what Friston calls “active inference.” Mathematically, the quality of this model corresponds to model evidence; namely the probability of sensory fluctuations under the model. In this setting, free energy provides a function of sensory states that must decrease when model evidence increases. In other words, self-organization—and implicitly any form of homoeostasis—can be cast as minimizing free energy (or, more simply, self-evidencing).

One must add that if the self-organizing system at issue is a nervous system, then—odd as this may sound—it is important to recognize that all other bodily systems (e.g., the viscera) are “external” to the nervous system^36^. Nervous systems sense, represent and act upon all other bodily systems (both vegetative and sensory-motor ones) in just the manner I have described. Nervous systems co-evolved with the other systems due to increasing complexity of organisms, which (complexity) requires orchestration of the multiple homeostatic demands arising from the various systems. Nervous systems are therefore meta-systems, performing meta-homeostatic functions on behalf of the entire body. Homeostatic regulation of the organism as a whole is delegated, as it were, to the nervous system.

In summary, homeostasis is explained by the causal dynamics mandated by the very existence of Markov blankets; in terms of which self-organizing systems generate a type of work that binds free energy and maintains the system in its typically occupied (“preferred” or “valued”) states. The concept of preferred states of self-organizing systems is identical with the concept of homeostatic settling points. The mathematical formulations quantifying the relevant dynamics of self-organizing systems need not be reproduced here (see Friston, 2013); since they concern the prerequisites of life in general rather than those for *consciousness* in particular. I will introduce the equations that are critical for our purposes in the next subsection.

Hopefully it is clear from the forgoing that although I have used quasi-physiological terms like “sensory” and “motor,” and quasi-psychological ones like “knowing,” “inference,” “belief,” “value” and “prediction,” the actual mechanisms I have described are simultaneously physiological *and* psychological ones. This (their abstract ontology) is their primary virtue, in light of what I said in section ‘The Problem With the Hard Problem’. As we shall now see, the very same abstractions can be extended to explain the function of consciousness in both its (psychological and physiological) manifestations. Indeed, that is why one is justified to use quasi-physiological and quasi-psychological terms for these mechanisms.

Now we come to the crux of the matter.

### How Does Consciousness Arise?

I first expressed the view in 1997 that the problem of consciousness will only be solved if we reduce its psychological and physiological manifestations to a single underlying abstraction (Solms, 1997)^37^. It took me many years to realize that this abstraction revolves around the dynamics of free energy and uncertainty (Solms, 2013, 2014).

Free energy minimization is the basic function of homeostasis, a function that is performed by the same brainstem nuclei that I was led to infer—like others, on independent (clinico-anatomical) grounds—were centrally implicated in the generation of consciousness. In other words, the functions of homeostasis and consciousness are realized physiologically in the very same part of the brain. This insight led to the collaborative work that enabled Friston and me to expand the variational free energy formulation of the mechanism of homeostasis to explain the mainspring of consciousness itself (Solms and Friston, 2018)^38^.

Readers may have noticed already that the dynamics of a Markov blanket generate two fundamental properties of minds—namely (elemental forms of) *selfhood* and *intentionality*. It is true that these dynamics also generate elemental properties of bodies—namely an *insulating membrane* (the ectoderm of complex organisms, from which the neural plate derives) and *adaptive behavior*. This is a remarkable fact. It underpins dual-aspect monism.

Section ‘In the Beginning Was the Affect’ focused mainly on the anatomy and physiology of homeostasis; now we are also clarifying its psychology, by explicating the deeper mechanism. Foundational to what we call psychology is the *subjective* observational perspective. The fact that self-organizing systems must monitor their own internal states in order to persist (that is, to exist, to survive) is precisely what brings *active* forms of subjectivity about. The very notion of selfhood is justified by this existential imperative. It is the origin and purpose of mind.

Selfhood is impossible unless a self-organizing system monitors its internal state in relation to not-self dissipative forces. The self can only exist in contradistinction to the not-self. This ultimately gives rise to the philosophical problem of other minds. In fact, the properties of a Markov blanket *explain* the problem of other minds: the internal states of a self-organizing system can only ever register hidden external (not-system) states vicariously, via the sensory states of their own blanket.

We have seen that minds emerge in consequence of the existential imperative of self-organizing systems to monitor their own internal states in relation to potentially annihilatory, entropic forces^39^. Such monitoring is an inherently value-laden process. It is predicated upon the biological ethic (which underwrites the whole of evolution) to the effect that survival is “good.” This imperative is formalized in terms of free-energy minimization.

Such negentropic dynamics of self-organizing systems are the absolute precondition for the evolution of minds. However, there is nothing about these dynamics which distinguishes conscious from unconscious mental processes. Put differently, there is nothing about such proto-mental dynamics which explains the emergence of feeling, as opposed to the exigencies of life. It is true that the dynamics described above revolve around value, but the values in question could—in principle—still be expressed in purely quantitative terms (e.g., 10 > 9). There is no necessity to introduce qualitative terms into the dynamics of free energy minimization.

What is it then, that underwrites the transition from unconscious (quantitative, “proto-mental”) states to conscious (qualitative, truly “mental”) ones? It seems the transition revolves fundamentally around increasing complexity. This refers to complexity of a specific type, however, not just complexity of integrated information processing in general (cf. Tononi, 2012). On the self-evidencing view, complexity acquires a very specific meaning^40^ (This follows from the fact that model evidence is the difference between accuracy and complexity. As model evidence is actively increased by minimizing free energy, the accuracy of predictions rises, with a concomitant increase in complexity. In other words, increasing model complexity is always licensed by an ability to make more accurate predictions).

Organisms evolve increasing self-complexity—for obvious adaptive reasons—as they diversify into (divide vegetative labor between) multiple sub-systems. For example, they evolve digestive vs. respiratory vs. thermoregulatory vs. immune systems. Each such specialized system is governed by a homeostatic imperative of its own. Metabolic energy balance, oxygenation, hydration, and thermoregulation (for example) are not the same things, although each of them contributes to the overall imperative of organism-wide free energy minimization. If the differential demands of the specialized homeostatic systems are going to be computed differentially (as they must) then it follows that increasing complexity requires some form of compartmentalization of quantities. Such compartmentalization can only be achieved through some form of *qualitative* differentiation between the sets of variables (e.g. 10 × X is worth more than 10 × Y; where X and Y are *categorical variables*). One can think of this compartmentalization as being something akin to a “color coding” or “flavoring” of the different data sets. This manifests in many different guises; from functional specialization in neuronal systems through to factorization of fundamental constructs that we use to model the world (e.g., “what” and “where” systems in the brain). As noted above, model evidence is the difference between accuracy and complexity, which requires increases in complexity to be nuanced (cf. Ockham's principle). Compartmentalization enables a simpler representation of what's going on “out there” in terms of external or non-self-states. Crucially, this sort of compartmentalization is essential for models that generalize to new situations.

In other words, the requirement for compartmentalization becomes a necessity when the relative value of the different quantities *changes* over time. For example: hunger trumps fatigue up to a certain value, whereafter fatigue trumps hunger; or hunger trumps fatigue in certain circumstances, but not others (i.e., 10 × X is currently [but not always] worth more than 10 × Y). Such changes require the system not only to compartmentalize its work efforts in relation to its different needs, but also to *prioritize* them over time.

This imperative reaches its nadir in the active states of the system, which inevitably produce a bottleneck. For example, organisms cannot eat and sleep simultaneously. Likewise, they cannot turn left and right at the same time. When it comes to action, executive choices must be made.

All these *contextual* factors become more prescient when one considers also how organisms survive in novel (unpredicted) environments. It is conceivable that an extremely complex set of algorithms could evolve (no matter how unwieldy they may become) to compute relative survival demands in all predictable situations, and to prioritize actions on this basis. But how does the organism choose between X and Y when the consequences of the choice are unpredictable? The physiological considerations discussed in the previous section suggest that it does so by *feeling* its way through the problem, where the direction of feeling (pleasure vs. unpleasure)—in the relevant modality—predicts the direction of expected uncertainty (decreasing vs. increasing)—within that modality^41^.

In selecting the best course of action, we must call upon our model of the world to predict the consequences of some behavior in terms of the expected free energy. *Expected free energy just is uncertainty about the consequences of any putative action*. The imperative to minimize expected free energy therefore becomes necessary to choose actions that minimize uncertainty and realize familiar, preferred sensory states.

Before we consider what this might entail in formal, mathematical terms, I want to make clear that the evolutionary considerations we have just reviewed suggest a *graded* transition from proto-mental to mental states (i.e., from unconscious to conscious subjectivity). Subjective values (i.e., system-centric values) are computed at the level of autonomic homeostasis already. This implies a potential for hedonic valence. But the qualitatively felt aspect of hedonic value does not *have to* be registered by the self-organizing system until multiple such values must be differentially computed and prioritized in variable and novel contexts, where uncertainty itself becomes the primary determinant of action selection.

Computationally, such contextual factors are formalized in terms of precision-weighting. “Precision” is an extremely important aspect of active and perceptual inference; it is the *representation of uncertainty*. The precision attaching to a quantity estimates its reliability, or inverse variance (e.g., visual—relative to auditory—signals are afforded greater precision during daylight vs. night-time). Heuristically, precision can be regarded as the confidence afforded probabilistic beliefs about states of the not-system—or, more importantly, what actions “I should select.”

This is the fundamental point made in Solms and Friston (2018). We were led to the conclusion that—whereas homeostasis requires nothing more than ongoing adjustment of the system's active states (M) and/or inferences about its sensory states (ϕ), in accordance with its predictive model (ψ) of the external world (Q) or vegetative body (Qη), which can be adjusted automatically on the basis of ongoing registrations of prediction error (e), quantified as free energy (F)—the contextual considerations just reviewed require an additional capacity to adjust the precision weighting (ω) of all relevant quantities. This capacity provides a formal (mechanistic) account of voluntary behavior—of choice.

With the above quantities^42^ in place, one can describe any self-organizing (i.e., self-evidencing) system with the following dynamics:

(1a) $$\begin{array}{rcl} \frac{\partial }{\partial t}M & = & -\frac{\partial F}{\partial M}=-\frac{\partial F}{\partial e}\frac{\partial e}{\partial M}=\frac{\partial \Phi }{\partial M}\cdot \omega \cdot e \end{array}$$

(1b) $$\begin{array}{rcl} \frac{\partial }{\partial t}Q & = & -\frac{\partial F}{\partial Q}=-\frac{\partial F}{\partial e}\frac{\partial e}{\partial Q}=-\frac{\partial \psi }{\partial Q}\cdot \omega \cdot e \end{array}$$

(1c) $$\begin{array}{rcl} \frac{\partial }{\partial t}\omega & = & -\frac{\partial F}{\partial \omega }=\frac{1}{2}\cdot \left(\omega^{-1}-e\cdot e\right) \end{array}$$

Where free energy and prediction error are:

(2) $$\begin{array}{rcl} F & = & \frac{1}{2}\cdot \left(e\cdot \omega \cdot e-\log\left(\omega \right)\right) \end{array}$$

(3) $$\begin{array}{rcl} e\; & = & \;\Phi \left(M\right)\;-\;\psi \left(Q\right) \end{array}$$

A more detailed account of the thinking behind these broad-brushstroke equations can be found in Solms and Friston (2018) and in the background references contained therein.

Physiologically, precision is usually associated with the *postsynaptic gain* of cortical neurons reporting prediction errors. This is precisely the function of ERTAS modulatory neurons (see section In the Beginning Was the Affect). In this sense, precision can be associated—through free energy minimization—with *selective arousal* (and thus, as formalized by the three dependencies in equation 1, with action [1a], perception [1b], and affect [1c], respectively).

It is useful to appreciate that every prediction error neuron (or neuronal population) is equipped with a specific—and changing—postsynaptic gain, and thereby with an implicit representation of precision. Precision is not a single value; every sensation and action—and every hierarchical abstraction, including every prediction and ensuing error signal—must be equipped with a precision which has to be optimized.

From the above equations, it is also clear that precision (consciousness) *controls* the influence of prediction errors on action (motivation) and perception (attention). Conceptually, precision is a key determinant of free energy minimization and the enabling—or activation—of prediction errors. In other words, precision determines which prediction errors are selected and, ultimately, how we represent the world and our actions upon it.

In this sense, precision plays the role of Maxwell's daemon^43^–selecting the passage of molecules (i.e., sensory signals) to confound the Second Law of thermodynamics. In this analogy, consciousness is nothing more or less than the activity of Maxwell's daemon (i.e., the optimization of precision with respect to free energy). That is, in this analogy, consciousness does not correspond to the passage of molecules that are enabled by the daemon (i.e., the perceptual sequelae of message passing in cortical hierarchies) but rather to the activity of the daemon itself.

This distinction is what underlies the prejudice (of Koch and others) to the effect that neuromodulation merely “enables” conscious content. The conceptual breakthrough reported here revolves around the insight that the residual error in each action/perception cycle (registered in PAG, see section ‘In the Beginning Was the Affect’) is *felt* uncertainty—i.e., that each of the various categories (or flavors) of error possess affective “content” of their own. Here, unpleasure (within the modality at issue) means *increasing uncertainty* in the modality, and pleasure means that *things are turning out as expected*. This (felt uncertainty) causally determines the (ERTAS) adjustments of subsequent sensory-motor priorities and expectations (i.e., of ω). That is, it determines selective arousal. This is the heart of the matter.

Note that this proposal calls on the notion of *activating* expectations or representations in the sense that—in the absence of precision—prediction errors could fail to induce any neuronal response. In other words, without precision, prediction errors could be sequestered at the point of their formation in the sensory epithelia (or at whichever level in the predictive processing hierarchy they occur). Physiologically, these sorts of states are encountered every day; for example, in stereotyped behavioral automatisms and during sleep (Hobson, 2009; Hobson and Friston, 2014)^44^.

The distinction between *interoceptive* and *exteroceptive* precision is central to this argument. If brains are sympathetic organs of inference, assimilating exteroceptive (sensory/motor) and interoceptive (vegetative) data through prediction, then their respective precision is about something (c.f. Brentano, 1874).

The proposal is that interoceptive precision is prioritized because the probabilistic beliefs attaching to what Panksepp calls homeostatic affects (e.g., hunger, thirst, sleepiness) cannot be overridden. Organismic beliefs at this level of the hierarchy are dictated by the phenotype, not by experience. This implies that everything which follows in the hierarchy, leading from the centrencephalic core to the sensorimotor periphery, is subordinated to affect. That is why I describe the adjustment of ω *per se* as “affect”. Consciousness *itself* is affective. Everything else (from motivation and attention, leading to action and perception, and thereby to learning)—all of it—is a functional of affect. Affect *obliges* the organism to engage with the outside world, and it thereby determines all of its active, subjectively embodied engagement with it.

None of this can go on in the dark.

Introspective precision is inherently about selfhood and intentionality (and therefore survival). Its compulsive quality is gradually diluted as the centrifugal processing hierarchy is traversed, through instinctual and sensory affective mechanisms, and the non-declarative behavioral stereotypes associated with them, via the declarative LTM systems, to the ever-changing STM periphery (see section ‘Consciousness Arises Instead of a Memory-Trace’ below).

The affective value implicit in ω must be an inherent property of any self-organizing system that proactively and contextually resists the Second Law of thermodynamics. Precision optimization determines the extent to which this value will be felt (i.e., expressed via selective enabling of belief updating) for purposes of choice. To be clear: it is easy to envision an organism (or machine) in which precision values are set in such a way that the system's responses to prediction error are automatized. Indeed, large swathes of the human nervous system (not to mention the rest of the body) are organized in this way.

It is noteworthy that qualitative fluctuations in affect (i.e., ω) arise continuously from periodic comparisons between the sensory states that were predicted—based upon a generative model of the internal body (*Q*η) and the world (ψ*(Q*) and samples of the actual sensory states (ϕ). This recurrent assessment of sensory states only gives rise to changes in subjective quality when the amplitude of prediction errors *changes*—signaling a change in uncertainty about the state of affairs and, in particular, the expected consequences of action (*M*). For this reason alone, it must be said—as one of my reviewers helpfully asked me to clarify—the Nirvana that the ideal self-organizing system described here strives for can never be attained in a real biological system, for the simple reason that change (both external and internal) always happens^45^.

Below, we will briefly consider the relation of this capacity to neural plasticity. It is difficult to conceive of a complex self-organizing system adapting flexibly to changing and novel environments in the absence of some such capacity. This, in my view, is how and why consciousness arises.

## “Consciousness Arises Instead of a Memory-trace”

This section will be disproportionately short (see Solms, 2018a,d, in press, for fuller treatments).

We saw above that conscious self-states are fundamentally affective states. Consciousness—in its most elementary form—is a sort of alarm mechanism, which guides the behavior of self-organizing systems as they negotiate situations beyond the bounds of their preferred states, in so far as they are not equipped with automatized (or automatable) predictions for dealing with them.

I explained in section ‘In the Beginning Was the Affect’ that the predictions which return us complex organisms to our preferred states are provided, in the first instance, by instinctual behaviors—which are innate survival tools. These tools serve us well, and are utilized willy nilly, but they cannot possibly do justice to the complexities of the environmental niches we actually find ourselves in. For this reason, innate predictions must be supplemented through learning from experience.

That is why we *feel* instinctual emotions: we feel them because they do not and cannot predict all the variance. What we feel, in short, is the residual prediction error and associated uncertainty as we surf unpredicted situations. This (feeling within a particular modality) guides the choices which—over time—generate new, acquired predictions, in the manner described in section ‘In the Beginning Was the Affect’.

But the ideal of such emotional learning is to automatize the acquired predictions (Some of them, such as fear conditioning, are automatized at the outset; others, like attachment bonding, are consolidated over longer time periods). Naturally, we need to forge new predictions which are at least as reliable as the innate ones, and to the extent that we achieve this (i.e., to the extent that prediction errors wane), to that extent acquired emotional predictions are automatized through consolidation, right down to the level of procedural memory systems (which are “hard to learn and hard to forget,” see Squire, 2004). In this way, the acquired predictions come to resemble the instinctual ones, not only in their functional properties^46^ but also in their subcortical anatomical localization.

The most important functional property of non-declarative memories is the very fact that they are non-declarative. This boils down to the fact that subcortical memory traces cannot be retrieved in the form of *images*, for the simple reason that they do not consist in cortical mappings of the sensory-motor surface organs^47^. They entail simpler cause-and-effect links of the kind that were described above as “associative learning of the connection between actions and their effects.”

The cortical (declarative) memory systems, by contrast, are always ready, on the basis of prediction errors, to revive the mental images they represent. In other words, declarative systems readily return LTM traces to the STM state of *conscious* working memory—in order to update them^48^. This necessarily entails activation (i.e., selection) of salient cortical representations—their salience being determined (and “flavored”) by the relevant prediction errors and variance. This process (which Friston calls “surprise”) should not be confused with the sensory affect of surprise. The felt affect in question may be *any* of the homeostatic, emotional or sensory affects.

It is important to note that felt affects typically incorporate both the selected error signal *and* the ensuing adjustment of cortical (and over longer time frames, subcortical) precisions. But as the latter (cognitive) component of predictive-work-in-progress binds the former (affective) free energy, so the conscious states in question will resemble conscious thinking rather than feeling^49^. Even conscious thinking requires the presence of a subject of experience, but the process becomes unconscious just as soon as it possibly can. This coincides neatly with the fact that feeling only persists (is only required) for as long as the cognitive task at hand remains unresolved. Conscious cognitive capacity is an extremely limited resource (cf. Miller's law, above) which must be used sparingly.

In these few words, we have explained the conscious part of cognition—the part that is left over “when the performance of all the [other] functions is explained” (Chalmers).

It is hopefully clear from the foregoing that the essential task of cognitive (cortical) consciousness is to *delay* motor responses to affective “demands made upon the mind for work^50^.” This delay enables thinking. The essential function of cortex is thus revealed to be stabilization of non-declarative executive processes—thereby raising them to a higher “cathectic level” (i.e., the bound state)—which is the essence of what we call (for good reason) *working* memory.

The above-described reversal of the consolidation process (*reconsolidation*; Nader et al., 2000) renders LTM-traces labile, through literal dissolution of the proteins that initially “wired” them (Hebb, 1949). This iterative feeling and re-feeling one's way through declarable problems is—on the proposal presented here—the function of the cognitive qualia which have so dominated contemporary consciousness studies. In short, conscious reconsolidation is predictive-work-in-progress. One is reminded of Freud (1920) obscure dictum: “consciousness arises instead of a memory-trace” (i.e., a labile trace is not a trace, it is a state of what Freud called drive “discharge”; see Solms, 2015).

Perceptual/cognitive consciousness (activated via attention), no less than affect itself, is a product of uncertainty. Non-declarative (subcortical) memory-traces are far less uncertain—more precise but also less complex—than declarative (cortical) ones. The relative degree of precision typically attaching to cortical vs. subcortical vs. autonomic prediction errors, therefore, coincides with the relative plasticity (resistance to change) of their associated beliefs.

One need only add that the exteroceptive sensory-motor modalities are “flavored” by consciousness in just the same way as interoceptive ones are, and for the same reason. This facilitates compartmentalization of the relevant data (and thereby reduces computational complexity) while the self-system surfs uncertainty in contextually variable conditions (The role of precision weighting in these conditions, in relation to the various perceptual modalities, and—most interestingly—in relation to language and inner speech, are discussed at length by Hohwy, 2013 and Clark, 2016).

These laconic formulations provide the basis for a new, integrated theory of affective and cognitive consciousness (and the unconscious).

## Conclusion

In this paper, I have drawn attention to two impediments to solving the “hard problem” of consciousness—one philosophical and one scientific—and I have suggested how these impediments might be removed. The first is the popular idea that the brain “produces” consciousness, i.e., that physiological processes literally *turn into* experiences, through some curious metaphysical transformation. The second impediment is the conventional notion that consciousness is a function of cerebral cortex, i.e., that visual awareness (or any other form of conscious cognition) serves as the model example of consciousness. Adopting a dual-aspect monist position on the philosophical mind/body problem allows us to find the causal mechanism of consciousness not in the manifest brain but rather in its *functional organization*, which ultimately underpins both the physiological and the psychological manifestations of experience. In order to transcend the figurative language of dualism, this unifying (monist) organization should be described in *abstract* terms (i.e., neither in physiological nor psychological terms but rather in mathematical ones). ‘Against this background,’ I (like Damasio and others) suggest that the long-sought mechanism of consciousness is to be found in *an extended form of homeostasis*, which describes the mode of functioning of both the deep brainstem nuclei that provide the NCC of affective arousal and the experience of feeling itself (which appears to be the foundational form of consciousness). This type of homeostasis (formalized here as free-energy minimization) entails the generation of affects (formalized as homeostatic prediction errors) which must be contextually prioritized in relation to each other and not-system events (formalized as precision weighting), leading to modulation of perception and action (formalized as error correction) on the basis of felt uncertainty. This modulatory arousal process, in turn, leads to *learning from experience* through reconsolidation, which bestows an enormous adaptive advantage over simpler types of homeostasis—such as those found in autonomic (involuntary) nervous systems and refrigerators—the advantage being a capacity for life-preserving intentional behavior in unpredicted situations.

## Author Contributions

The author confirms being the sole contributor of this work and has approved it for publication.

### Conflict of Interest Statement

The author declares that the research was conducted in the absence of any commercial or financial relationships that could be construed as a potential conflict of interest.

## Footnotes

^1^Freud was a dual-aspect monist (see Solms, 1997). Here, I am disregarding the clinical complexities arising from the developmental achievement of recognizing oneself in the mirror.

^2^When Freud first introduced this term (Letter to Fliess of March 10, 1898; Freud, 1950 [1892-99]) he said it refers to a level of explanation that incorporates both psychology and biology. In this way he aspired to “transform metaphysics into metapsychology” (Freud, 1901, p. 259).

^3^Freud's priority in formulating this “functionalist” position is not recognized: “[We] attempt to make the complications of mental functioning intelligible by *dissecting the function* and assigning its different constituents to different component parts of the apparatus. So far as I know, the experiment has not hitherto been made of using this method of dissection in order to investigate the way in which the mental instrument is put together, and I can see no harm in it.” (Freud, 1900, p. 536, emphasis added).

^4^Freud put it like this: “We should picture the instrument which carries out our mental functions as resembling a compound microscope or photographic apparatus, or something of the kind. On that basis, psychical locality will correspond to a point inside the apparatus at which one of the preliminary stages of an image comes into being. In the microscope and telescope, as we know, these occur at ideal points, regions in which no tangible component of the instrument is situated” (Freud, 1900, p. 536).

^5^This was the goal of the Helmholtz school of medicine: “Brücke and I pledged a solemn oath to put into effect this truth: ‘No other forces than the common physical and chemical ones are active within the organism. In those cases which cannot currently be explained by these forces one has either to find the specific way or form of their action by means of the physical-mathematical method or to assume new forces equal in dignity”’ (Du Bois-Reymond, 1842; Letter to Hallmann, in Du Bois-Reymond, 1918). The young Freud was a pupil of the Helmholtz school, and described Brücke as one of his formative role-models (Freud, 1925a).

^6^Freud always insisted that ‘unconscious affect’ is an oxymoron: “It is surely of the essence of an emotion that we should be aware of it, i.e., that it should become known to consciousness. Thus, the possibility of the attribute of unconsciousness would be completely excluded as far as emotions, feelings, and affects are concerned” (Freud, 1915a, p. 177). He explains: “The whole difference arises from the fact that ideas are cathexes—basically of memory-traces—whilst affects and emotions correspond to processes of discharge, the final manifestations of which are perceived as feelings. In the present state of our knowledge of affects and emotions we cannot express this difference more clearly” (ibid., p. 178).

^7^Strachey called Freud's distinction between ‘quotas of affect’ and ‘memory-traces of ideas’ the “most fundamental of all his hypotheses.” (Strachey (1962), p. 63)

^8^At that time, “mind” and “consciousness” were synonymous. Despite his many disagreements with Meynert, Freud endorsed the view that consciousness is nothing more than “a sense organ for the perception of psychical qualities” (1900, p.615) and, moreover, that this “sense organ” was located in the *cerebral cortex*: “We have merely adopted the views on localization held by cerebral anatomy, which locates the ‘seat’ of consciousness in the cerebral cortex—the outermost, enveloping layer of the central organ. Cerebral anatomy has no need to consider why, speaking anatomically, consciousness should be lodged on the surface of the brain instead of being safely housed somewhere in its inmost interior.” (Freud, 1920, p. 24). This cortical localization applied even to the affective aspect of consciousness (see Freud, 1940, pp, 161-2).

^9^Ironically, in light of Freud's comment cited above, consciousness is *not* located “on the surface of the brain [but is instead] safely housed somewhere in its inmost interior.” (Freud, 1920, p. 24)

^10^Cf. Meynert (1884) assertion: “The motor effects of our consciousness reacting upon the outer world are not the result of forces innate in the brain. The brain, like a fixed star, does not radiate its own heat: it obtains the energy underlying all cerebral phenomena from the world beyond it” (English trans., p. 160). Freud's views on this important point vacillated (see Solms and Saling, 1990).

^11^It will likewise enable us to approach the young Freud (1950 [1895]) unrequited aspiration “to represent psychical processes as quantitatively determinate states”. Cf. his earlier remark to the effect that quotas of affect “possess all the attributes of a quantity (*though we have no means of measuring it*), which is capable of increase, diminution, displacement and discharge” (Freud, 1894, p. 60, emphasis added).

^12^The reasonable criterion here must be the same as it is for any other scientific question, namely, are predictions from the hypothesis (that these animals and children are conscious) *disconfirmed* or not? see (Panksepp et al., 2016).

^13^Of course, this does not imply that other structures do not also participate, even (and importantly) including some beyond the confines of the nervous system. The function of affect is being ‘localized’ in the conventional sense demanded by Teuber's “double-dissociation” paradigm, which states that if function A is lost with damage to structure X but not structure Y, and function B is lost with damage to structure Y but not structure X, then functions A and B are two independent functions. (Here A = consciousness; B = cognition; X = brainstem; Y = cortex).

^14^Freud shared the cortico-centric view that even affects are felt only when the underlying ‘psychical energies’ arouse what he termed the ‘inner surface’ of the system Pcpt.-Cs. in the cerebral cortex (see footnote 8 above)

^15^This view was not original; it coincided almost exactly with Freud's view to the effect that “oscillations in the tension of instinctual needs […] become conscious as feelings in the pleasure-unpleasure series” (1940, p. 198). Damasio (1999) acknowledged Freud's priority.

^16^There is no generally-agreed-upon definition of ‘instinct’ but it should be noted that the term is being used here in the mainstream biological sense rather than the Freudian one (which, incidentally, arose from a mistranslation of the German term Trieb; see Solms, 2018c).

^17^Many commentators forget that the term “homeostasis” was only introduced into biology in 1926. Freud conceptualized the same function as “drive.” In this respect, the following extract from Solms (2013, pp. 79-80) serves as a summary of the present article: “I define drive as ‘a measure of the demand made upon the mind for work in consequence of its connection with the body” (Freud, 1915a, p. 122), where the “measure” is the degree of deviation from a homeostatic set-point (with implications for survival and reproductive success). I do not believe that this deviation itself is something mental, but the ‘demand’ it generates is felt in the pleasure-unpleasure series. This (felt demand) is affect, which in my view is the origin of mind. The “work” that flows from affect is cognition, the functional purpose of which is to reduce affect—that is, to reduce prediction error (free energy). The purpose of cognition is to bring the world into line with our predictions and our predictions into line with the world. This centrally involves learning.”

^18^Damasio (2018) attributes feeling states only to creatures with nervous systems (see Solms, 2018b).

^19^In the view on offer here, therefore, unlike Freud's, the drive *is* the feeling (Solms, 2013). Drive literally brings the mind into being. Before the drive is felt it is not a drive – it is simple homeostasis, which can be regulated by autonomic reflexes and behavioral stereotypes. The present view also differs from Freud's in conceptualizing pleasure-unpleasure as deviations to and from a settling point, as opposed to Freud's continuum, and in conceptualizing Nirvana as that settling point rather than something ‘beyond’ the pleasure principle (see Solms, 2018b).

^20^The conflicting demands of the different needs that these many “flavors” represent underpins mental conflict, and (equally importantly) accounts for the many behaviors one sees in nature—and in psychopathology—which are by no means obviously “self-preservative.”

^21^Panksepp (1998) and Merker (2007) provide cogent evidence for the view that this prioritization process pivots around a midbrain “decision triangle” (Panksepp calls it the “SELF”) whereby *needs* are registered in the periaqueductal gray (PAG) and *opportunities* in the superior colliculi.

^22^Cf. Freud's notion that thinking is interposed between drive and action. The contents of this paragraph are necessarily overly dense. These highly complex issues require more space than a journal article allows. See Solms, in press, for a more detailed explication.

^23^I say ‘typically’ because foraging is commonly the most adaptive response to contextual uncertainty. However, all manner of other instincts may be selected, which are so conditioned through learning from experience, that they are frequently no longer recognizable as instincts at all. (Cf. what is said below about learning in relation to the SEEKING instinct, which serves as a model example.)

^24^This seems to be identical with Freud (1950 [1895]) conception of the cognitive effects of ‘experiences of satisfaction’; i.e., wishful cathexis, etc. For the role played by opioids in such experiences, see Berridge et al. (2009). Panksepp (1998) and Schultz (2016) offer distinctly different accounts of the role played by dopamine.

^25^Freud endorsed the concept of basic emotions, although he classified them differently from how we do today, and he conflated them with his conception of primal phantasy—which entailed the untenable notion of inherited episodic memories. See Freud, 1916-17, p. 395.

^26^Focal lesions of the PAG produce persistent vegetative states, and DBS there elicits powerful affects of various kinds—not only negative ones—depending upon which part of the PAG is stimulated.

^27^See footnote 21 above. Freud (1900), too, placed the system Cs. at the motor end of the apparatus, but he evidently had *cortical* motor mechanisms in mind.

^28^Freud (1900), too, pictured a functional overlap between Cs. interoception and Pcpt. exteroception, and eventually he combined the two systems under the single rubric “Pcpt.-Cs.” (Freud, 1917). However, once again, he clearly had *cortical* systems in mind.

^29^I have emphasized the physiological considerations over the psychological ones in this account. The parallel commentary in these footnotes draws attention to the fact that the physiological inferences we have reached strongly resemble the psychological inferences that Freud was led to. For him, feelings (the pleasure principle) were the bedrock of mental life—including cognition.

^30^Freud (1920) encapsulated this fundamental biophysical dynamic in his second drive theory. Before that, he formulated it as a compromise—the “constancy principle”—which he imagined as being effected by a reticulum of “constantly cathected neurons” (Freud, 1950 [1895]), the “great reservoir” of his later “ego,” the “bound energy” of which gave negentropic power to the “secondary process.” By this I mean the capacity to inhibit neuronal discharge (called “freely mobile energy” in Freud's terminology), which he equated with the action of the Second Law (see his principle of “neuronal intertia,” the direct ancestor of the “death drive”). In Friston's predictive processing framework, this same negentropic power is attributed to predictive neuronal assemblies (which are directly equivalent to Freud's LTM Ψ neurons) which inhibit transmission of sensory signals—Freud's STM Φ neurons—thereby minimizing “prediction error” and all the entropic perturbations it gives rise to, measured as “free energy”. Cf. (Carhart-Harris and Friston, 2010).

^31^Cf. Freud's concepts: (i) “Q screens” or “stimulus barriers”, (ii) “ϕ neurons” or “system Pcpt” and (iii) “M neurons” or “system Cs,” respectively. Incidentally, most Freud scholars do not seem to realize that Q, in thermodynamics, quantifies heat.

^32^“Ergodicity” is a statistical property, whereby the average of any measurable function of a random dynamical system *converges* over a sufficient period of time. In short, dynamical systems that possess measurable characteristics over periods of time must be (nearly) ergodic.

^33^A “Markov blanket” induces a statistical partitioning of internal and external states, and *hides* the latter from the former. The Markov blanket itself consists in two sets (“sensory” and “active” states) which influence each other in a circular fashion: external states cause sensory states which influence—but are not influenced by—internal states, while internal states cause active states which influence—but are not influenced by—external states.

^34^Freud would have called such predictions “unconscious phantasies.”

^35^This sensory sampling process is reminiscent of Freud's image of the system Ucs periodically palpating the system Pcpt-Cs with cathectic feelers (Freud, 1925b, p. 231). ‘Beliefs’, in the sense used here, are taken to be probability distributions whose parameters or sufficient statistics correspond to system states.

^36^Freud (1950 [1895]) speaks of “the somatic element itself” generating Q (which he designates Qη) by virtue of “an increasing complexity of the interior of the organism.” (p. 297).

^37^Freud's unifying abstraction was the “mental apparatus”. The philosophical implications of his oft-repeated insistence that the instrument of the mind is unconscious “in itself” are not sufficiently appreciated (see Wakefield, 2018). Hence his laconic remark: “the unconscious is the proper mediator between the somatic and the mental, perhaps the long-sought ‘missing link”’ (letter to Georg Groddeck dated June 5, 1917; see Groddeck, 1977).

^38^When we did so, I experienced something similar to what Freud described more than a century before, when he wrote: “Everything seemed to fit together, the gears were in mesh, the thing gave one the impression that it was really a machine and would soon run of itself […] Of course, I cannot contain myself with delight.” (Letter to Fliess of October 20, 1895; Freud, 1950 [1892-99]).

^39^Cf. Freud's formulation of narcissism (“hate, as a relation to objects, is older than love"; Freud (1915b), p. 139) which became the foundation of Melanie Klein's ‘paranoid schizoid position’.

^40^Technically, it is the relative entropy between posterior and prior beliefs or probability distributions over external or not-self states.

^41^A common source of confusion here is the fact that the dopaminergic SEEKING modality (discussed in Section ‘In the Beginning Was the Affect’) engages *positively* with uncertainty. Its innate non-declarative prediction translates as: ‘engagement with a source of uncertainty provides maximal opportunities to resolve that uncertainty’. Therefore, in the case of this instinct, lack of engagement with uncertainty is “bad” (cf. anergia, abulia, anhedonia, hopelessness). The conceptual distinction in the affective neuroscience of our time between “appetitive” and “consummatory” pleasures removes the source of Freud's puzzlement in his lifelong attempts to establish a psychophysics of pleasure-unpleasure in relation to oscillations in the tension of drive needs.

^42^ω, precision; ψ, prediction; ϕ, perception; *M*, action; *Q*, {inferred] world; *F*, free energy; *e*, prediction error. Psychoanalytic readers will recognize some of these quantities from Freud, 1950 [1895]). We use the same symbols in recognition of the penetrating insights contained in his “Project,” although it has become necessary—in line with some further insights recorded in the footnotes above—to use them slightly differently from what Freud had in mind.

^43^Maxwell's daemon is a thought experiment created by James Clerk Maxwell to suggest how the second law of thermodynamics might be violated: in brief, a daemon controls a small door between two chambers of gas. As gas molecules approach, the daemon opens and shuts the door, so that fast molecules pass to the other chamber, while slow molecules remain in the first, thus decreasing entropy.

^44^It could easily be argued that this same mechanism – i.e. setting precision values so that prediction errors induce no response – underpins repression (see Solms, 2018a). This is what Freud's notion of repression as “a failure of translation” amounts to within the present framework (Letter to Fliess, December 6, 1896; Freud, 1950 [1892-99]).

^45^As the Talking Heads song poetically tells us: “Heaven is a place / where nothing ever happens.”

^46^Cf. (Freud's, 1915a) “special characteristics of the system Ucs,” all of which can be reduced to the functional characteristics of the procedural and emotional memory systems (see Solms, 2018d).

^47^Cf. (Freud's, 1923) notion of the “bodily ego” being derived from cortical projections of the sensory-motor periphery.

^48^This property of declarative LTMs coincides exactly with what Freud called the system Pcs, although in my view the Pcs consists in both word and thing presentations (both semantic and episodic traces). Surely, there are no thing presentations in the Ucs (in non-declarative memory), only stereotyped action programmes.

^49^This corresponds roughly to Freud's distinction between freely mobile and bound cathexes. However, we should not overlook the fact that the *goal* of thinking is automatization. Bound cathexes are, in short, merely *tolerated* by the ego (cf. Freud's compromise “constancy principle”). The ego's *ideal* state remains Nirvana (a curious state in which there is no residual free energy and precision becomes infinite).

^50^This coincides exactly with Freud's notion of “secondary process.” Freud described the distinction between free and bound nervous energy as his “deepest insight” and added: “I do not see how we can avoid making it.” (Freud, 1915a, p. 188)

## References

[B1] Bargh J. Chartrand T. (1999). The unbearable automaticity of being. Am. Psychol. 54, 462–479. 10.1037/0003-066X.54.7.462

[B2] Berridge K. (1996). Food reward: brain substrates of wanting and liking. Neurosci. Biobehav. Rev. 20, 1–25. 10.1016/0149-7634(95)00033-B 8622814

[B3] Berridge K. Robinson T. Aldridge J. W. (2009). Dissecting components of reward: ‘liking’, ‘wanting’, and learning. Curr. Opin. Pharmacol. 9, 65–73. 10.1016/j.coph.2008.12.014 19162544 PMC2756052

[B4] Blomstedt P. Hariz M. Lees A. Silberstein P. Limousin P. Yelnik J.. (2007). Acute severe depression induced by intraoperative stimulation of the substantia nigra: a case report. Parkinsonism Relat. Disord. 14, 253–256. 10.1016/j.parkreldis.2007.04.005 17561432

[B5] Brentano F. (1874). Psychology From an Empirical Standpoint. London: Routledge.

[B6] Carhart-Harris R. Friston K. (2010). The default mode, ego functions and free energy: a neurobiological account of Freudian ideas. Brain 133, 1265–1283. 10.1093/brain/awq010 20194141 PMC2850580

[B7] Chalmers D. (1995). Facing up to the problem of consciousness. J. Consciousness Stud. 2, 200–219.

[B8] Chalmers D. J. (1996). The Conscious Mind: In Search of a Fundamental Theory. Oxford: Oxford University Press.

[B9] Clark A. (2016). Surfing Uncertainty. Oxford: Oxford University Press 10.1093/acprof:oso/9780190217013.001.0001

[B10] Craig A. D. (2009). How do you feel – now? The anterior insula and human awareness. Nat. Rev. Neurosci. 10, 59–70. 10.1038/nrn2555 19096369

[B11] Crick F. (1994). The Astonishing Hypothesis. New York, NY: Scribner.

[B12] Damasio A. (1994). Descartes' Error. New York, NY: Putnam.

[B13] Damasio A. (1999). Commentary to Panksepp, emotions as viewed by psychoanalysis and neuroscience. Neuropsychoanalogy 1, 38–39. 10.1080/15294145.1999.10773242

[B14] Damasio A. (2010). Self Comes to Mind. New York, NY: Pantheon.

[B15] Damasio A. (2018). The Strange Order of Things. New York, NY: Pantheon.

[B16] Damasio A. Carvalho G. (2013). The nature of feelings: evolutionary and neurobiological origins. Nat. Rev. Neurosci. 14, 143–152. 10.1038/nrn3403 23329161

[B17] Damasio A. Damasio H. Tranel D. (2012). Persistence of feeling and sentience after bilateral damage of the insula. Cereb. Cortex 23, 833–846. 10.1093/cercor/bhs077 22473895 PMC3657385

[B18] Du Bois-Reymond E. (1918). Letter to Hallmann, 1842. Jugendbriefe von Emil Du Bois-Reymond an Eduard Hallmann. Berlin: Dietrich Reimer. p. 108.

[B19] Ellis G. Solms M. (2018). Beyond Evolutionary Psychology: How and Why Neuropsychological Modules Arise. Cambridge: Cambridge University Press.

[B20] Freud S. (1893). Charcot, Standard Edn. Vol. 3 London: Hogarth Press p. 11–23.

[B21] Freud S. (1894). The Neuro-Psychoses of Defence, Standard Edn. Vol. 3 London: Hogarth Press p. 45–61.

[B22] Freud S. (1900). The Interpretation of Dreams, Standard Edn. London: Hogarth Press, 4 and 5.

[B23] Freud S. (1901). The Psychopathology of Everyday Life. Standard Edn. London: Hogarth Press p. 6.

[B24] Freud S. (1915a). The Unconscious, Standard Edn. Vol. 14 London: Hogarth Press p. 166–204.

[B25] Freud S. (1915b). Instincts and Their Vicissitudes, Standard Edn. Vol. 14 London: Hogarth Press p. 117–140.

[B26] Freud S. (1916-17) Introductory Lectures in Psychoanalysis, Standard Edn. London: Hogarth Press p. 15–16.

[B27] Freud S. (1917). Metapsychological Supplement to the Theory of Dreams. Standard Edn. Vol. 14 London: Hogarth Press p. 222–235.

[B28] Freud S. (1920). Beyond the Pleasure Principle, Standard Edn. Vol. 18 London: Hogarth Press p. 7–64.

[B29] Freud S. (1923). The Ego and the id. Standard Edn. Vol. 19 London: Hogarth Press p. 12–59.

[B30] Freud S. (1925a). An Autobiographical Study, Standard Edn. London: Hogarth Press. p. 20.

[B31] Freud S. (1925b). A Note Upon “the mystic writing-pad,” Standard Edn. Vol. 16 London: Hogarth Press p. 227–232.

[B32] Freud S. (1940). An Outline of Psychoanalysis, Standard Edn. Vol. 23 London: Hogarth Press p. 144–207.

[B33] Freud S. (1950 [1892-99]). Extracts From the Fliess Papers, Standard Edn. Vol. 1 London: Hogarth Press p. 174–280.

[B34] Freud S. (1950 [1895]) Project for a Scientific Psychology. Standard Edn. Vol. 1 London: Hogarth Press p. 281–397.

[B35] Friston K. (2013). Life as we know it. J. R. Soc. Interface 10:20130475. 10.1098/rsif.2013.0475 23825119 PMC3730701

[B36] Golaszewski S. (2016). Coma-causing brainstem lesions. Neurology 87:2433. 10.1212/WNL.0000000000003417 27815403

[B37] Groddeck G. (1977). The Meaning of Illness: Selected Psychoanalytic Writings Including His Correspondence With Sigmund Freud. London: Hogarth.

[B38] Harlow J. M. (1868). Recovery from the passage of an iron bar through the head. Publicat. Massachusetts Med. Soc. 2, 327–347.

[B39] Hebb D. (1949). The Organization of Behavior. New York, NY: John Wiley.

[B40] Hobson J. A. (2009). REM sleep and dreaming: towards a theory of protoconsciousness. Nat Rev Neurosci. 10, 803–813. 10.1038/nrn2716 19794431

[B41] Hobson J. A. Friston K. (2014). Consciousness, dreams, and inference: the Cartesian theatre revisited. J. Consciousness Stud. 21, 6–32.

[B42] Hohwy J. (2013). The Predictive Mind. Oxford: Oxford University Press 10.1093/acprof:oso/9780199682737.001.0001

[B43] Huston J. Borbely A. (1974). The thalamic rat: general behaviour, operant learning with rewarding hypothalamic stimulation, and effects of amphetamine. Physiol. Behav. 12, 433–448. 10.1016/0031-9384(74)90121-8 4820139

[B44] Kandel E. Schwartz J. Jessell T. Siegelbaum S. Hudspeth A. (2012). Principles of Neural Science, 5th Edn New York, NY: Elsevier.

[B45] Kihlstrom J. (1996). Perception without awareness of what is perceived, learning without awareness of what is learned, in The Science of Consciousness: Psychological, Neuropsychological and Clinical Reviews, ed, Velmans M. (London, Routledge), p. 23–46.

[B46] Koch C. (2004). The Quest for Consciousness. New York, NY: WH Freeman.

[B47] LeDoux J. Brown R. (2017). A higher-order theory of emotional consciousness. Proc. Natl.Acad. Sci. U.S.A. 114, E2016–E2025. 10.1073/pnas.1619316114 28202735 PMC5347624

[B48] Levine J. (1983). Materialism and qualia: the explanatory gap. Pac. Philos. Q. 64, 354–361. 10.1111/j.1468-0114.1983.tb00207.x

[B49] Merker B. (2007). Consciousness without a cerebral cortex: a challenge for neuroscience and medicine. Behav. Brain Sci. 30, 63–134. 10.1017/S0140525X07000891 17475053

[B50] Meyer J. Quenzer L. (2005). Psychopharmacology: Drugs, The Brain, and Behavior. Sunderland, MA: Sinauer Associates.

[B51] Meynert T. (1884). Psychiatrie. Klinik der Erkrankungen des Vorderhirns, begründet auf dessen Bau, Leistungen und Ernährung, Vienna: Wilhelm Braumüller.

[B52] Mongillo G. Barak O. Tsodyks M. (2008). Synaptic theory of working memory. Science 319, 1543–1546. 10.1126/science.1150769 18339943

[B53] Moruzzi G. Magoun H. (1949). Brain stem reticular formation and activation of the EEG. Electroencephalog. Clin. Neurol. 1, 455–473. 10.1016/0013-4694(49)90219-9 18421835

[B54] Munk H. (1878). Weitere Mittheilungen zur Physiologie der Grosshirnrinde. Arch für Physiol. 2, 162–177

[B55] Munk H. (1881). Ueber die Funktionen der Grosshirnrinde. Berlin: August Hirschwald.

[B56] Nader K. Schafe G. E. Le Doux J. (2000). Fear memories require protein synthesis in the amygdala for reconsolidation after retrieval. Nature 406, 722–726. 10.1038/35021052 10963596

[B57] Nagel T. (1974). What is it like to be a bat? Philos. Rev. 83, 435–450. 10.2307/2183914

[B58] Panksepp J. (1998). Affective Neuroscience. Oxford: Oxford University Press.

[B59] Panksepp J. Lane R. D. Solms M. Smith R. (2016). Reconciling cognitive and affective neuroscience perspectives on the brain basis of emotional experience. Neurosci. Biobehav. Rev. 76, 187–215. 10.1016/j.neubiorev.2016.09.010 27640756

[B60] Parvizi J. Damasio A. (2003). Neuroanatomical correlates of brainstem coma. Brain 126, 1524–1536. 10.1093/brain/awg166 12805123

[B61] Penfield W. Jasper H. (1954). Epilepsy and the Functional Anatomy of the Human Brain. Oxford: Little & Brown.

[B62] Pfaff D. (2006). Brain Arousal and Information Theory. Cambridge, MA: Harvard University Press 10.4159/9780674042100

[B63] Pulsifer M. Brandt J. Salorio C. Vining E. Carson B. Freeman J. (2004). The cognitive outcome of hemispherectomy in 71 children. Epilepsia 45, 243–254. 10.1111/j.0013-9580.2004.15303.x 15009226

[B64] Schultz W. (2016). Dopamine reward prediction error coding. Dialogues Clin. Neurosci. 18, 23–32. 27069377 10.31887/DCNS.2016.18.1/wschultz PMC4826767

[B65] Searle J. (1997). The Mystery of Consciousness. New York, NY: New York Review of Books.

[B66] Searle J. (2017). Foreword, in Biophysics of Consciousness: A Foundational Approach. eds Poznanski R. Tuszynski J. Feinberg T. (New York, NY: World Scientific) 129–148.

[B67] Sharma J. Angelucci A. Sur M. (2000). Induction of visual orientation modules in auditory cortex. Nature 404, 841–847. 10.1038/35009043 10786784

[B68] Shewmon D. Holmse D. Byrne P. (1999). Consciousness in congenitally decorticate children: developmental vegetative state as a self-fulfilling prophecy. Dev. Med. Child Neurol. 41, 364–374. 10.1017/S0012162299000821 10400170

[B69] Solms M. (1997). What is consciousness? [and response to commentaries]. J. Amer. Psychoanal. Assn. 45, 681–778. 10.1177/00030651970450031201 9353705

[B70] Solms M. (2013). The conscious id. [and response to commentaries]. Neuropsychoanalysis 15, 5–85 10.1080/15294145.2013.10773711

[B71] Solms M. (2014). A neuropsychoanalytical approach to the hard problem of consciousness. J. Integr. Neurosci. 13, 173–185. 10.1142/S0219635214400032 25012708

[B72] Solms M. (2015). Reconsolidation: turning consciousness into memory. Behav. Brain Sci. 38, 40–41. 10.1017/S0140525X14000296 26050688

[B73] Solms M. (2018a). What is ‘the unconscious’ and where is it located in the brain? Ann. NY Acad. Sci. 1406, 90–97. 28759698 10.1111/nyas.13437

[B74] Solms M. (2018b). Review of damasio, the strange order of things. J. Am. Psychoanal. Ass. 66, 579–586. 10.1177/0003065118780182

[B75] Solms M. (2018c). Extracts from the revised standard edition of Freud's complete psychological works. Int. J. Psychoanal. 99, 11–57. 10.1080/00207578.2017.1408306 33951842

[B76] Solms M. (2018d). The neurobiological underpinnings of psychoanalytic theory and therapy. Front. Behav. Neurosci. 12:294. 10.3389/fnbeh.2018.00294 30564106 PMC6288296

[B77] Solms M. (in press). Consciousness Itself: Feeling and Uncertainty. London: Profile Books.

[B78] Solms M. Friston K. (2018). How and why consciousness arises: some considerations from physics and physiology. J. Conscious. Stud. 25, 202–238.

[B79] Solms M. Kaplan-Solms K. Brown J. W. (1996). Wilbrand's case of ‘mind blindness’, in Classic Cases in Neuropsychology, eds Code C. Joanette Y. Lecours A. Wallesch C.-W. (London: Psychology Press), 89–110.

[B80] Solms M. Panksepp J. (2012). The 'id' knows more than the 'ego' admits. Brain Sci. 2, 147–175. 10.3390/brainsci2020147 24962770 PMC4061793

[B81] Solms M. Saling M. (1990). A Moment of Transition: Two Neuroscientific Articles by Sigmund Freud. London: Karnac.

[B82] Squire L. (2004). Memory systems of the brain: a brief history and current perspective. Neurobiol. Learn. Mem. 82, 171–177. 10.1016/j.nlm.2004.06.005 15464402

[B83] Strachey J. (1962). The Emergence of Freud's Fundamental Hypotheses. Standard Edn (London: Early Psycho-Analytic Publications), 3, 62–68.

[B84] Teuber H.-L. (1955). Physiological psychology. Ann. Rev. Psychol. 6, 267–296. 10.1146/annurev.ps.06.020155.001411 14377367

[B85] Tononi G. (2012). Phi: A Voyage from the Brain to the Soul. New York, NY: Pantheon.

[B86] Wakefield J. (2018). Freud and Philosophy of Mind, Volume 1: Reconstructing the Argument for Unconscious Mental States. London: Palgrave Macmillan 10.1007/978-3-319-96343-3

